# Augmented Reality-Assisted Ultrasound Breast Biopsy

**DOI:** 10.3390/s23041838

**Published:** 2023-02-07

**Authors:** Nuno Costa, Luís Ferreira, Augusto R. V. F. de Araújo, Bruno Oliveira, Helena R. Torres, Pedro Morais, Victor Alves, João L. Vilaça

**Affiliations:** 1 2Ai—School of Technology, IPCA, 4750-810 Barcelos, Portugal; 2 Algoritmi Center, School of Engineering, University of Minho, 4800-058 Guimaraes, Portugal; 3 LASI—Associate Laboratory of Intelligent Systems, 4800-058 Guimaraes, Portugal; 4 Institute of Computing, Universidade Federal Fluminense (UFF), Niteroi 24210-310, Brazil; 5 Life and Health Sciences Research Institute (ICVS), School of Medicine, University of Minho, 4710-057 Braga, Portugal; 6 ICVS/3B’s—PT Government Associate Laboratory, 4710-057 Braga/Guimaraes, Portugal

**Keywords:** ultrasound, breast biopsy, augmented reality, convolutional neural networks, lesion segmentation

## Abstract

Breast cancer is the most prevalent cancer in the world and the fifth-leading cause of cancer-related death. Treatment is effective in the early stages. Thus, a need to screen considerable portions of the population is crucial. When the screening procedure uncovers a suspect lesion, a biopsy is performed to assess its potential for malignancy. This procedure is usually performed using real-time Ultrasound (US) imaging. This work proposes a visualization system for US breast biopsy. It consists of an application running on AR glasses that interact with a computer application. The AR glasses track the position of *QR* codes mounted on an US probe and a biopsy needle. US images are shown in the user’s field of view with enhanced lesion visualization and needle trajectory. To validate the system, latency of the transmission of US images was evaluated. Usability assessment compared our proposed prototype with a traditional approach with different users. It showed that needle alignment was more precise, with 92.67 ± 2.32° in our prototype versus 89.99 ± 37.49° in a traditional system. The users also reached the lesion more accurately. Overall, the proposed solution presents promising results, and the use of AR glasses as a tracking and visualization device exhibited good performance.

## 1. Introduction

The leading cause of death worldwide is cancer [1]. Breast cancer is the world’s most prevalent cancer, excluding nonmelanoma skin cancers, and the fifth leading cause of cancer death, with 2.3 million women diagnosed and 685,000 deaths worldwide in 2020 [2]. Though sizeable portions of breast cancer cases do not result in death, they significantly reduce the quality of life and generally come with higher expenses.

The treatment of this type of cancer is more effective in its early stages. As a result, medical imaging modalities such as mammography and ultrasound (US) are used to screen substantial parts of the population [3,4]. When mammography or US finds a suspicious lesion, a biopsy is performed to evaluate the nature of the lesion. A biopsy consists of taking a small sample of body tissue so it can be examined in a laboratory to determine its potential for malignancy. Currently, real-time US-guided biopsy is the preferred method to perform a breast biopsy [5].

US imaging is a non-invasive, radiation-free technique that effectively captures tissue characteristics by transmitting sound waves into the body and recording the waves that echo back to construct an image. It requires relatively simple technology and has no known harmful effects on health. In ultrasound, since the image is dynamically acquired and relies on the 3D motion of the probe, operator expertise is critical during acquisition; poor image quality and eventual artifacts further increase the demand on the operator’s expertise [6]. In conventional clinical practice, lesion identification in US is based purely on images through a naked-eye evaluation. Contouring lesions can be performed to extract relevant information, but it takes time and depends on the observers [7]. As a result, several methods have been suggested for automatic lesion segmentation. Active contour, region-growth, and threshold-based techniques were the main segmentation techniques used in early research. These techniques, however, frequently require manual intervention [8]. Recently, convolutional neural networks (CNN) have surpassed traditional image processing techniques and are showing promising results in breast US segmentation [9,10].

Subpar quality in US imaging can cause the performing physician to miss the lesion [11]. Furthermore, the sampling of lesion tissue can also have a maximum error rate of 10%, according to Heil et al. [12]. The biopsy procedure is, therefore, highly dependent on the skills of the physician. This work aims to improve this procedure by utilizing CNN lesion segmentation to help analyze US images by incorporating augmented reality (AR), that provides relevant information, such as the real-time tracking of the biopsy needle, in the field of view.

AR-based imaging systems may be helpful in the biopsy procedure. Essentially, these systems work by superimposing digital data over the user’s field of view (captured by the camera), creating the illusion that holographic content is a part of the real world. They can also utilize cameras to track the movements of objects and display information along those movements, removing the need for the user to look away from the object’s motion [13].

The integration of US-guided breast biopsy with AR systems was initially attempted in 1996 by Fuchs et al. [14]. In this initial work, the results were promising, and the developed system was sufficiently robust and accurate. Feedback from physicians reported that it helped the procedure; however, it had a lengthy setup process and no needle guidance. Due to technological limitations, the AR device weighed nearly six pounds. That work was continued by Rosenthal et al. [15] in 2002. They used more advanced AR glasses and implemented needle trajectory. More recently, in 2020, Asgar-Deen et al. [16] compared the usage of AR to a traditional monitor in a simple stationary breast biopsy procedure. In 2021, Gouveia et al. [17] utilized AR in magnetic resonance-assisted breast cancer surgery. In the same year Cattari et al. [18] developed an AR visualization tool for US-guided interventions. However, it relies on already existing volumetric US data, meaning the system lacks US real-time feedback. In the same year, Guo et al. [19] developed a system for renal biopsy training. This system uses *QR* codes to locate a mannequin, place a 3D rendering of the internal organs, a US probe, and the puncture needle to provide the best puncture path for the user. In 2022, Kashiwagi et al. [20], Nguyen et al. [21] and our previous work [22] experimented with AR glasses as a visualization tool for US images. These solutions only display the US images in the user’s field of view. This work places the US image with lesion location information in the correct position according to the real position and orientation of the US probe.

The objective of this work is to conceive a solution to train or increase the efficiency of physicians with the US-guided breast biopsy procedure. This paper describes a system prototype that utilizes Microsoft’s HoloLens 2 AR glasses for the enhancement of the US-guided breast biopsy procedure. It combines real-time US video, CNN segmentation, and object tracking to provide an enhanced visualization of the procedure. Moreover, the system’s latency is measured by comparing the developed system with an optical tracking device and the usability assessment of different users.

Overall, the current work introduces two novelties. The first is a new AR-assisted ultrasound breast biopsy system with embedded AI for lesions in ultrasound imaging, consequently allowing real-time detection and localization of possible lesions in the three-dimensional world of the AR glasses. The second novelty is the assistance for the biopsy needle alignment, which helps the user to position the needle at the correct alignment so that it shows in the generated US image.

The work is structured as follows. Section 2 presents how the proposed system was developed, followed by Section 3, which explains the experiments to assess usability and latency and its results. Section 4 discusses the achieved results. Finally, Section 6 presents the main conclusions.

## 2. Materials and Methods

### 2.1. Overview

**Figure 1:**
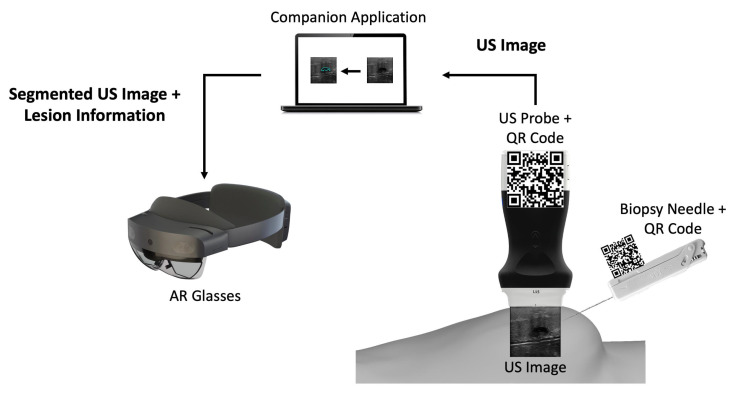
Overview of the different components of the proposed prototype.

The developed prototype uses a portable US probe, more specifically the Clarius L15 HD Portable Handheld Scanner, an automatic biopsy needle, the HoloLens 2 glasses, a computer, and two 7 cm wide *QR* codes mounted on 3D printed cases (Figure 1). The system is divided into an application running in AR glasses and a computer application.

The computer application is a companion to the AR application. It works by: (i) connecting to the US probe; (ii) processing the real-time incoming US images by segmenting them through a CNN network, and (iii) sending those images to the AR application afterwards.

The AR application running on the glasses is mainly a visualization tool. It tracks two *QR* code tags mounted on the US probe and the biopsy needle, so it can perform real-time tracking of both. It displays in the user’s field of view the real-time US video stream along with a trajectory from the needle tip to the lesion and validates its orientation.

### 2.2. Companion Application

The necessity for real-time US imaging is crucial in the biopsy procedure. However, US imaging presents poor quality and requires a high level of expertise to be interpreted [23]. In light of this, the solution must show real-time US video and provide a more straightforward US imaging interpretation.

Incompatibilities and limitations with the AR glasses system led to the development of a computer companion application that feeds data to the AR application. These data consist of the real-time streaming of US video from the US probe’s API along with the CNN lesion segmentation data. The CNN segmentation required an NVIDIA Graphics Processing Unit (GPU) since it relied on its CUDA API [24]. Since the AR system was lacking such a GPU, this computation had to be performed on a computer. The abovementioned CUDA API makes use of the parallelism available in a GPU [25,26], such as CNN operations, to speed up computations [27]. This application was developed using Qt (version 5.15) to create the Graphical User Interface (GUI), and OpenCV (version 4.5.5) was utilized to conduct US image processing and lesion segmentation.

#### 2.2.1. US Breast Lesion Segmentation

**Figure 2:**
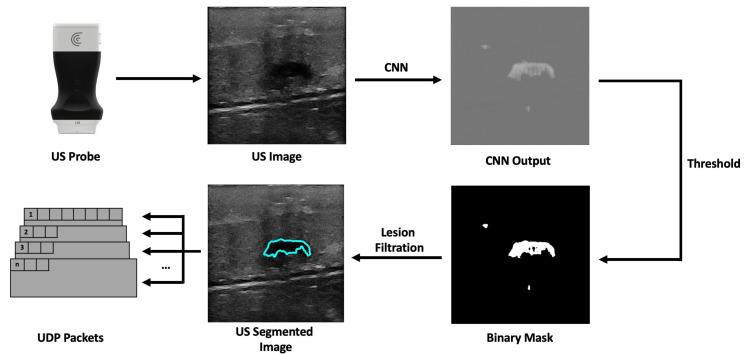
Companion application workflow overview.

The workflow of the companion application starts with the reception of a new US image frame from the US probe. This image has 512 × 512 pixels, dimensions manually set in the probe’s API. These dimensions were chosen so that the image would have the correct size to be used as input by the CNN model. The network architecture was U-Net, which performs well in tumor segmentation [28], based on the work developed with automatic lesion segmentation in breast cancer applications specialized in breast US images [29,30]. The incoming US image is converted, using the OpenCV library, from a 3-channel RGB image to a single-channel grayscale image before being passed to the network, which expects a 512 by 512-pixel wide grayscale image. The network’s output is a grayscale image the same size as the input that highlights what it considers to be lesions. Following that, a binary mask is created. Using a sigmoid function, the pixels’ values are normalized between 0 and 1. Then, pixels with values above a threshold of 0.9 are converted into white pixels, and pixels with values below it are converted into black pixels. This results in a segmentation mask where the segmented lesion is white within a black background. The generated binary mask could detect the lesions most of the time. However, it also identifies image noise as lesions. Fixing this issue was accomplished by calculating a bounding box encompassing every single lesion; the bounding boxes are then ordered by size, and the largest one is assumed to be the lesion. After that, the lesion’s position is calculated using its bounding box (Figure 2). The dimensions of the US image may vary in accordance with the ultrasound’s depth, but the streamed image dimensions remain the same, being filled with transparent pixels. However, we removed these transparent bands, ultimately decreasing the image’s width or height.

#### 2.2.2. Communication Protocol

The companion application sends the processed US image and additional information to the AR application. Therefore, a communication protocol was developed to bridge both applications. The choice regarding the communication protocol revolves around the use of Transmission Control Protocol (TCP) or User Datagram Protocol (UDP). Although stream-oriented TCP protocol is a robust protocol that provides reliability, flow control, and congestion control, it is somewhat slow in terms of performance. On the other hand, the UDP protocol is datagram-oriented, simpler than TCP, only guarantees the integrity of packets, and is much more useful for applications where low latency is essential [31,32]. So, the UDP protocol was chosen as the communication protocol for the task at hand. The datagram protocol devised consists of 512 byte-sized packets that have the US image data partitioned between them. It has (i) a header consisting of a 2-byte number that identifies the partition of the image data; (ii) an 8-byte US image frame identifier, and (iii) 1 byte identifying whether or not that packet is the last one. The receiving application uses the header information to determine when a packet is lost in the communication process. It does this by comparing the current packet number with the last packet number with the same image frame identifier. If the compared numbers are not sequential or the frame identifier is different, the packet sequence is dropped, and the application waits for the next first packet. In addition, the number one packet has additional meta-information about the US image, such as the width and height, the image’s pixel spacing, and the predicted lesion center in x and y-pixels.

### 2.3. AR Application

The original idea was, to use AR technology to help in the breast biopsy procedure by placing a hologram in the real-world location of the lesion. This would be achieved utilizing the tracking feature to locate the position of the US probe’s transducer and the automatic lesion prediction (given by the CNN network) to compute the lesion position to place a hologram there. With this information, the user would try to reach the virtual lesion displayed by the AR glasses. This approach, however, proved to be difficult to use. For starters, the breast might deform with the biopsy needle puncture, and the patient may breathe or do slight movements with the torso, possibly altering the position of the lesion. In addition, the AR glasses’ holograms also provide a misleading sense of depth, which became evident during tests. Hence, a real-time tracking of the biopsy needle was implemented, as well as a real-time display of the real-world position of the lesion. The application was developed for the HoloLens 2 glasses using Unity 3D 2021 (version 2021.3.2f1), Microsoft’s Mixed Reality Toolkit for Unity and Vuforia AR Software Development Kit (SDK).

#### 2.3.1. Needle and US Probe Tracking

**Figure 3:**
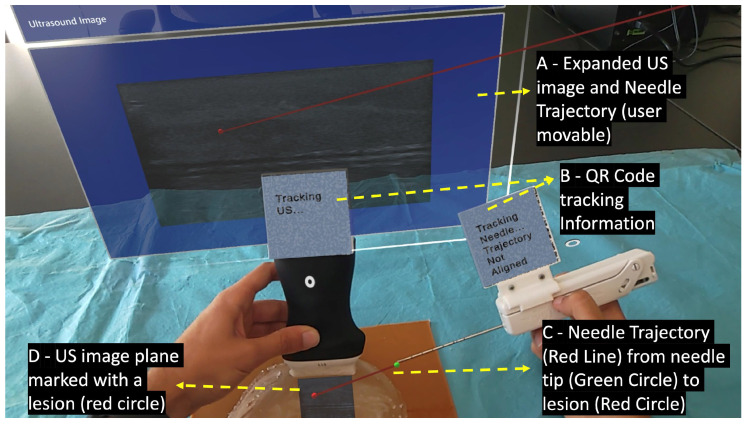
User’s point of view performing the biopsy procedure with the AR device.

Performing the biopsy procedure with the AR glasses (Figure 3) requires the localization of its main components, such as the US image, in the 3D world, along with the relative position of the biopsy needle tip. Therefore, the US probe and the biopsy needle require constant tracking by the AR glasses. The solution to actively track both objects revolved around tracking a physical *QR* code tag mounted on these objects. This is performed utilizing the Vuforia SDK to recognize *QR* code images in 3D space. The SDK needs prior information of the *QR* code image and its real-world size. The SDK uses this information to look for pre-determined features on the *QR* codes. Custom casings were 3D-printed to house the *QR* code tags on the US probe and on the biopsy needle. These casings were made of polylactic acid and were printed on the Ultimaker II 3D printer with a printing resolution of 0.2 mm.

When the Vuforia SDK is actively tracking a *QR* code, it places a virtual square hologram with the exact dimensions and orientation as the real *QR* code on top of it to inform the user whether the object is being actively tracked or not. The AR application uses the coordinates of the hologram to access the 3D world position of the *QR* code. These coordinates represent the center of the *QR* code tag in the virtual 3D world. By having the *QR* code tag’s center, the real-world size of the 3D printed casing, and the physical object it is mounted on, the application can precisely determine the location of the US probe’s transducer and the tip of the biopsy needle.

#### 2.3.2. AR Hologram Generation

**Figure 4:**
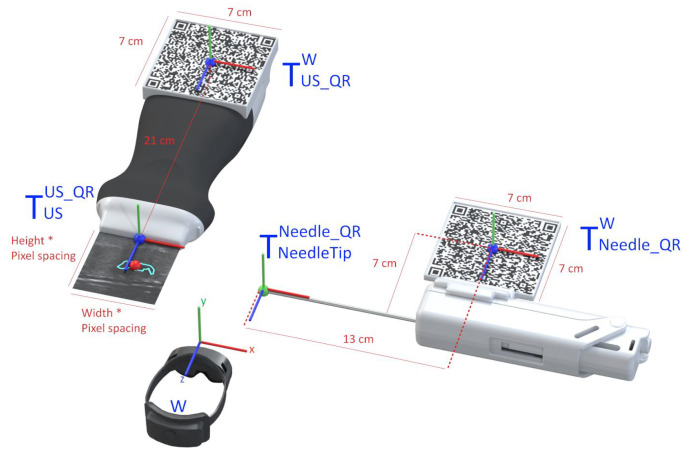
Representation of the conversion between the real-world objects and their virtual location.

The AR glasses use the *QR* code markers to track the pose of the needle and the US probe, which allows accurately placing the holograms in the 3D world. To accomplish this, we utilize the real dimensions of the *QR* codes, which the application knows before the tracking process, as references to estimate the target position and orientation. For it, we use the AR system as the reference coordinate system (i.e., the world), and in real-time, we estimate the transformations $T_{US\_QR}^{W}$ and $T_{Needle\_QR}^{W}$ that map the pose of the *QR* code attached to the ultrasound and needle, respectively, with the world (i.e., the AR system). Once we have these transformations, we compute the pose of the needle and ultrasound tip from the predefined fixed transformations $T_{US}^{US\_QR}$ and $T_{NeedleTip}^{Needle\_QR}$, respectively. Equations (1) and (2) show these conversions in more detail. Figure 4 depicts a visual representation of this conversion. In both cases, a CAD software was used to precisely measure the required offset between the centroid of the *QR* code and the device’s tip on the digital models (in STL format) of the US probe and the biopsy needle. The digital probe model was provided directly by the manufacturer. The outer surface of a real biopsy needle was manually designed in SolidWorks by two experienced designers.

(1) $$T_{US}^{W}=T_{US\_QR}^{W}\times T_{US}^{US\_QR}$$

(2) $$T_{NeedleTip}^{W}=T_{Needle\_QR}^{W}\times T_{NeedleTip}^{Needle\_QR}$$

The UDP packets received from the companion application contain various segments of a US image, that are later combined into a single image hologram. To match the real-world dimensions in the virtual environment, this hologram is resized according to the pose of the US probe. For it, the hologram’s dimensions are obtained by multiplying the image’s pixel spacing by its width and height. This hologram is then displayed in the correct location and orientation of the already calculated probe’s transducer. An expanded version of the hologram is also displayed to facilitate the visualization of small details of the US image. A red circle is also placed in the lesion center virtual coordinates inside the hologram showing the user where the predicted lesion is located.

**Figure 5:**
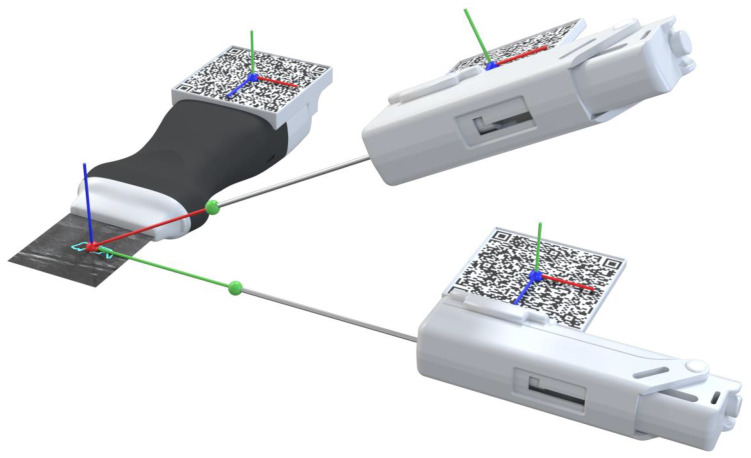
Needle alignment and calculation representation. Alignment is represented by the green line and unalignment is represented by thew red line.

The physician needs to visualize the needle in real-time while performing the ultrasound imaging to ensure that the needle will hit the targeted lesion [33]. In this work, strategies were implemented to assist the user in aligning the biopsy needle with the US image plane. This was accomplished by displaying a line from the tracked needle tip, as shown in Figure 4 (green ball), to the predicted lesion that changes colors depending on the needle’s alignment. The colors vary between green, which indicates alignment, and red, indicating unalignment, as shown in Figure 3 (red line) and Figure 5. The calculation of this alignment is based on the angle between two vectors: the vector between the needle tip and the lesion position and the normal vector of the US image hologram plane. The cross-product of these two vectors is calculated, and afterwards the resulting angle is computed, as described in [34]. The ideal angle value is precisely 90°, meaning that both vectors form a right angle. This value, however, is virtually impossible to reach in the application context due to hand movements and computational uncertainties. An angle range was, therefore, empirically defined, ranging between 86 and 94°.

Using the proposed system, the procedure would start by localizing the lesion in the US image with the help of the CNN segmentation. Then, the user must track the probe and the biopsy needle’s *QR* code tags so the system can calculate the alignment of both elements and guide the biopsy needle to the predicted lesion, using a virtual line than connects them.

## 3. Results

### 3.1. Latency and Execution Times

The described prototype relies on a real-time calculation to correctly function. Thus, an essential metric to evaluate in the system is latency. In particular, the latency between the US being generated in the US probe to that image being displayed in the AR system. Therefore, the experiment consisted of marking two distinct positions on a breast phantom and comparing the image’s timestamps to compare time differences.

**Figure 6:**
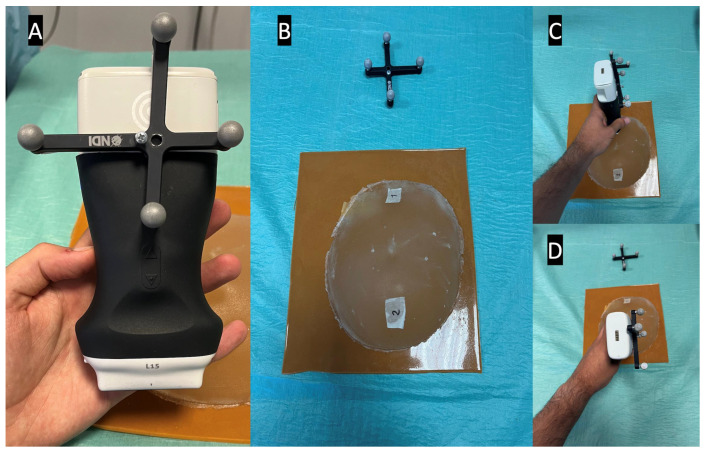
Latency test setup, showing in picture (**A**) the optical marker mounted on the US probe, in picture (**B**) the breast phantom with both positions marked, and in pictures (**C**,**D**) the US probe in its different test positions.

The NDI Polaris Vega ST optical tracker measurements were used as a benchmark to evaluate the latency. This tracker has a sampling rate of 60 Hz and maximum latency of 16 ms. The probe was fitted with an optical navigation marker (Figure 6A), and during the experiment, the coordinates of the probe, along with the timestamp of each position, were registered. Meanwhile, the AR glasses save the received US images and their timestamps. Since the US images acquired in the two positions are different, it is possible to measure the time difference between the last image of the first position and the first image of the second position (Figure 6). This time difference is compared with the optical tracker data to measure the latency of the AR image-displaying system. The latency values were recorded from 10 runs and their average was calculated. In addition, the costliest operations that account for the system’s latency were evaluated over 10 2-min runs.

**Figure 7:**
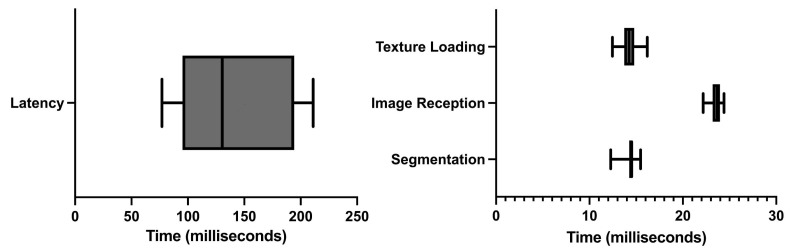
Latency and execution times.

The average time between moving the US probe and that movement being registered in the AR glasses was 143 ± 51.492 milliseconds (ms). Figure 7 shows the latency time data along with the execution times of the observed costliest operations.

Of all the different operations, three were observed to be costliest performance-wise: (i) the CNN segmentation of the US image; (ii) the render time of the US image on the AR glasses, and (iii) the time and failure rate in the reception of US images in the AR application. The longest execution time was of the reception of US images in the AR application. On average, it took 23.681 ± 0.637 ms, followed by the US image rendering on the AR application with 14.247 ± 0.953 ms. Finally, the segmentation computing took 14.341 ± 0.831 ms. It was also noted that, on average, 57.3% of the images sent were lost in the communication process.

### 3.2. Usability

**Figure 8:**
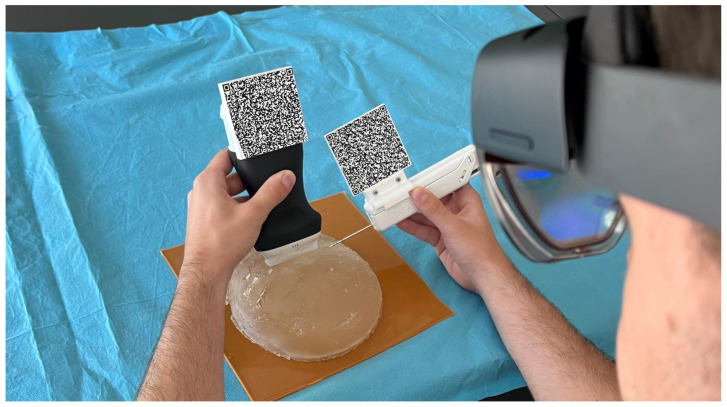
Third-person usability test view of a user performing the biopsy in a breast phantom.

The developed system prototype could assist either a novice or an experienced physician. Thus, its usability must be simple but effective in aiding the breast biopsy procedure. The assessment was made using 18 people, with a median age of 23.3 years and some or no experience with the biopsy procedure (Figure 8). A custom version of our system was developed, mimicking a traditional biopsy procedure, without any visualization enhancement. However, even in this version, the system needs to track both QR codes to measure the user’s accuracy in targeting what the user thinks is a lesion versus the CNN-predicted lesion.

Initially, the user was informed of how the biopsy procedure is performed and instructed to perform the procedure until they think they have reached the lesion. Then, the built-in calibration tool in the HoloLens glasses is used to calibrate the rendering engine for each user. Finally, the user performs the procedure with the limited version and developed prototype and the relevant data is stored on the device. Then, we evaluate the accuracy of the procedure, the time, the angle between the intersection of the US hologram plane normal, and the Euclidean distance between the lesion and needle tip location with all the gathered data.

**Figure 9:**
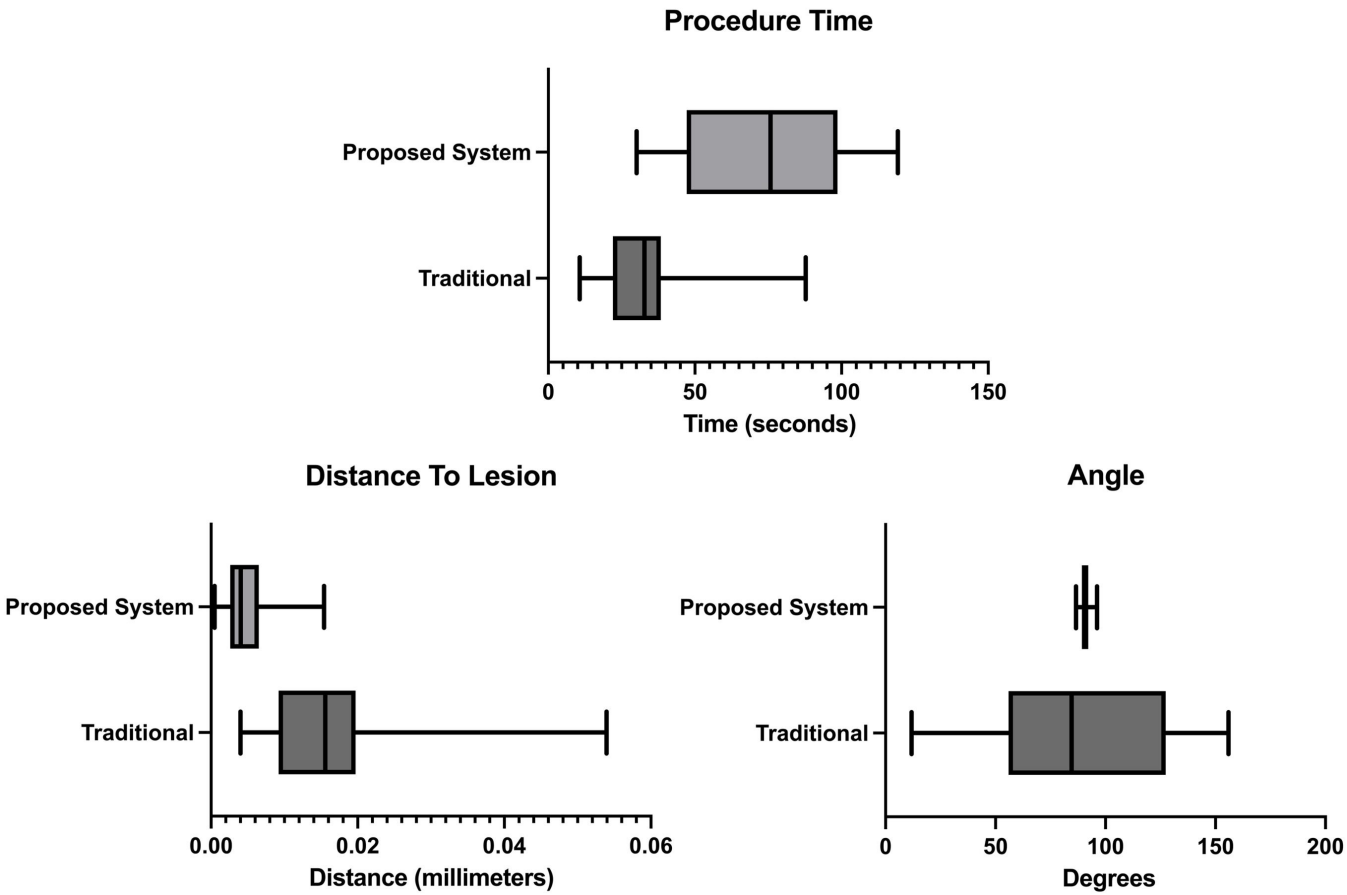
Procedure time, distance from the needle tip to the lesion and angle measurements.

The usability test results presented in Figure 9 show that the user, on average, claimed to have completed the biopsy procedure faster in the no-help traditional version, with a time of 33.5 s. Our proposed system with needle guidance and lesion prediction took an average of 74.1 s. However, the results demonstrate that the user reached the targeted lesion more regularly, with a much lower standard deviation. According to the results acquired, the procedure allowed the users to reach the predicted lesion location more accurately and precisely in our proposed system.

## 4. Discussion

According to the literature, similar solutions have been attempted. The most similar system was developed in 2002 by Rosenthal et al. [15]. This solution used dated equipment compared with today’s standards and did not have real-time lesion location prediction. It did, however, prove the concept of using AR as a valuable help in the procedure by having positive physician feedback. Other research has also attempted to use AR glasses as a visualization tool for US images, such as Kashiwagi et al. [20] or Nguyen et al. [21]. These approaches facilitated hand-eye coordination since users do not need to look away from the US probe’s movements to see the US video stream. To the best of our knowledge, no study has presented a technique that uses AR glasses to display real-time video from a US probe while simultaneously displaying lesion prediction from a CNN network and showing the trajectory of the biopsy needle in real time. This work is focused on proving the concept and technology for AR-assisted US-guided breast biopsies.

The results show that the developed prototype has a significant latency of 143 ms on average. This value is partly due to the data streaming from the companion application to the AR application via a wireless connection. According to the execution times tests, the computation that lasted longer was the reception of US frames, lasting approximately 23.681 ms. However, the failure rate in the reception of US image frames is 57.3%, meaning most frames sent via the network are lost. The approximate US frame reception time is significantly smaller than the latency value. This phenomenon can be attributed to the extra computation needed for rendering (14.247 ms) and CNN image segmentation (14.341 ms). The latency time measurements are performed by analyzing the received US image frames on the AR application and the timestamp in which they are received, comparing these with the data generated by the optical tracker. However, due to the high failure rate with the communication process, the exact image frame generated in the US probe’s second position might be lost, artificially increasing the latency times.

The usability assessment tests showed that the developed prototype improves the overall procedure as opposed to a traditional scenario. For starters, as shown in Figure 9-procedure time results, the measured time increases, meaning the users spend more time aligning the biopsy needle with the US image plane. The observed times are higher than the ones obtained in Bluvol et al. [35]; this may be due to the adaptation to the visualization in the AR glasses. The needle’s alignment with the US image became more precise, as noted by the much lower standard deviation value shown in the angle results in Figure 9. These results can be attributed to the implemented angle guidance, which helps the user insert the needle at the optimal angle. The distance between the tip of the needle to the estimated lesion position (distance to lesion results in Figure 9) decreased from 16.25 mm in the traditional version to 5.09 mm in the proposed version, so it is possible to assume that the process became more precise in the proposed version. The variation in the image segmentation can partially explain this difference. The distance is calculated from the needle tip to a single point that roughly indicates the center of the lesion. However, this point may vary due to slight variations in the US probe’s position. The custom no-help application is not the optimal comparison to the developed prototype, as the user needs to wear the AR glasses to track the position of the US probe and the biopsy needle (for comparison with the helped version). The user, however, needed to see the US images on an external display through the AR glasses screen. Possibly hampering the procedure by limiting the visibility of the US video. The system was also tested by a radiologist who gave positive feedback and pointed out that, in the future, it may be used as well as a training device.

As potential disadvantages of the proposed concept, we must say that the AR technology being used in this work suffers from the already mentioned misleading sense of depth that hampers the hologram visualization during the procedure. The image quality is also not ideal and can inflict some motion sickness on the user after long periods. Furthermore, since the device is wireless, it has limited performance that can increase latency and require external workstations to alleviate some of the performance load. Finally, the high cost of AR technology can further impede its adoption [36,37].

## 5. Study Limitations

Besides the improvement on the biopsy obtained with the proposed system, there are some limitations that should be addressed in the future. First, only Microsoft’s HoloLens 2 headset was used. In this aspect, the headset used provided every feature necessary to accomplish this. Still, in the future it would be interesting to test different headsets and see if the results are consistent.

Second, the calibration between the US image hologram and the US probe is not exact. This calibration is performed by capturing the probe’s *QR* code center and then rigidly transforming the tracked position to the bottom of the ultrasound (part in with the body surface). During the experiments of this work, no relevant errors were found when locating and reaching specific lesions displayed with the hologram. However, it may results in small misalignments, since the real sound beam generator site was not considered.

Third is the lack of clinical testing. The usability testing was conducted by non-clinicians with little or no experience with the breast procedure. With this being said, we received positive feedback from a radiologist.

## 6. Conclusions

This paper describes and evaluates a solution for the US-guided breast biopsy procedure. The AR glasses receive US images from a companion application along with lesion segmentation data on each image, which is then displayed in the user’s field of view. The application calculates the positions of the US probe’s transducer and the biopsy needle tip using the tracking data and the actual measurements of the objects. These data are combined into a single solution that displays needle tracking and lesion segmentation on the user’s field of view. The results show that the proposed solution improves the overall accuracy and precision of the procedure when compared to a traditional system.

This solution can potentially be used in clinical practice, possibly enhancing the information that a physician has in their line of sight. The system can also help the biopsy training process by providing needle alignment and trajectory guidance to trainees.

As for future work, a new tracking solution must be pursued to cut the reliance on *QR* codes, which somewhat limit usability during the biopsy procedure. An effort to use an optical tracker is contemplated. With the help of an optical tracker, it would be possible to know the location and orientation of objects, even when hidden from the user’s line of sight. The high latency measured and the high failure rate in the image streaming process must also be addressed by improving the communication protocol or experimenting with a wired connection. Additionally, clinical validation will be pursued.

## Data Availability

Not applicable.

## Abbreviations

The following abbreviations are used in this manuscript:

US	Ultrasound
CNN	Convolutional Neural Network
AR	Augmented Reality
GPU	Graphics Processing Unit
GUI	Graphical User Interface
TCP	Transmission Control Protocol
UDP	User Datagram Protocol
SDK	Software Development Kit

## References

[1] Lei S., Zheng R., Zhang S., Wang S., Chen R., Sun K., Zeng H., Zhou J., Wei W. (2021). Global patterns of breast cancer incidence and mortality: A population-based cancer registry data analysis from 2000 to 2020. Cancer Commun..

[2] WHO-GLOBOCAN Database. https://gco.iarc.fr/today/home.

[3] McKinney S.M., Sieniek M., Godbole V., Godwin J., Antropova N., Ashrafian H., Back T., Chesus M., Corrado G.S., Darzi A. (2020). International evaluation of an AI system for breast cancer screening. Nature.

[4] Wang L. (2017). Early Diagnosis of Breast Cancer. Sensors.

[5] Bick U., Trimboli R.M., Athanasiou A., Balleyguier C., Baltzer P.A.T., Bernathova M., Borbély K., Brkljacic B., Carbonaro L.A., Clauser P. (2020). Image-guided breast biopsy and localisation: Recommendations for information to women and referring physicians by the European Society of Breast Imaging. Insights Into Imaging.

[6] Hindi A., Peterson C., Barr R.G. (2013). Artifacts in diagnostic ultrasound. Rep. Med. Imaging.

[7] Hu Y., Guo Y., Wang Y., Yu J., Li J., Zhou S., Chang C. (2019). Automatic tumor segmentation in breast ultrasound images using a dilated fully convolutional network combined with an active contour model. Med. Phys..

[8] Jiménez-Gaona Y., Rodríguez-Álvarez M.J., Lakshminarayanan V. (2020). Deep-Learning-Based Computer-Aided Systems for Breast Cancer Imaging: A Critical Review. Appl. Sci..

[9] Wang K., Liang S., Zhang Y. (2021). Residual Feedback Network for Breast Lesion Segmentation in Ultrasound Image.

[10] Ayana G., Dese K., woon Choe S. (2021). Transfer Learning in Breast Cancer Diagnoses via Ultrasound Imaging. Cancers.

[11] Tagliabue E., Dall’Alba D., Magnabosco E., Tenga C., Peterlik I., Fiorini P. (2019). Position-based modeling of lesion displacement in ultrasound-guided breast biopsy. Int. J. Comput. Assist. Radiol. Surg..

[12] Heil J., Sinn P., Richter H., Pfob A., Schaefgen B., Hennigs A., Riedel F., Thomas B., Thill M., Hahn M. (2018). RESPONDER–diagnosis of pathological complete response by vacuum-assisted biopsy after neoadjuvant chemotherapy in breast Cancer-a multicenter, confirmative, one-armed, intra-individually-controlled, open, diagnostic trial. BMC Cancer.

[13] Mahmood F., Mahmood E., Dorfman R.G., Mitchell J., Mahmood F.U., Jones S.B., Matyal R. (2018). Augmented Reality and Ultrasound Education: Initial Experience. J. Cardiothorac. Vasc. Anesth..

[14] Fuchs H., State A., Pisano E.D., Garrett W.F., Hirota G., Livingston M., Whitton M.C., Pizer S.M. (1996). Towards Performing Ultrasound-Guided Needle Biopsies from Within a Head-Mounted Display.

[15] Rosenthal M., State A., Lee J., Hirota G., Ackerman J., Keller K., Pisano E.D., Jiroutek M., Muller K., Fuchs H. (2002). Augmented reality guidance for needle biopsies: An initial randomized, controlled trial in phantoms. Med. Image Anal..

[16] Asgar-Deen D., Carriere J., Wiebe E., Peiris L., Duha A., Tavakoli M. (2020). Augmented Reality Guided Needle Biopsy of Soft Tissue: A Pilot Study. Front. Robot. AI.

[17] Gouveia P.F., Costa J., Morgado P., Kates R., Pinto D., Mavioso C., Anacleto J., Martinho M., Lopes D.S., Ferreira A.R. (2021). Breast cancer surgery with augmented reality. Breast.

[18] Cattari N., Condino S., Cutolo F., Ferrari M., Ferrari V. (2021). In Situ Visualization for 3D Ultrasound-Guided Interventions with Augmented Reality Headset. Bioengineering.

[19] Guo Z., Tai Y., Du J., Chen Z., Li Q., Shi J. (2021). Automatically Addressing System for Ultrasound-Guided Renal Biopsy Training Based on Augmented Reality. IEEE J. Biomed. Health Inform..

[20] Kashiwagi S., Asano Y., Goto W., Morisaki T., Shibutani M., Tanaka H., Hirakawa K., Ohira M. (2022). Optical See-through Head-mounted Display (OST-HMD)–assisted Needle Biopsy for Breast Tumor: A Technical Innovation. In Vivo.

[21] Nguyen T., Plishker W., Matisoff A., Sharma K., Shekhar R. (2022). HoloUS: Augmented reality visualization of live ultrasound images using HoloLens for ultrasound-guided procedures. Int. J. Comput. Assist. Radiol. Surg..

[22] Costa J.N., Gomes-Fonseca J., Valente S., Ferreira L., Oliveira B., Torres H.R., Morais P., Alves V., Vilaca J.L. Ultrasound training simulator using augmented reality glasses: An accuracy and precision assessment study. Proceedings of the 2022 44th Annual International Conference of the IEEE Engineering in Medicine & Biology Society (EMBC).

[23] Tolsgaard M.G., Todsen T., Sorensen J.L., Ringsted C., Lorentzen T., Ottesen B., Tabor A. (2013). International Multispecialty Consensus on How to Evaluate Ultrasound Competence: A Delphi Consensus Survey. PLoS ONE.

[24] Garland M., Grand S.L., Nickolls J., Anderson J., Hardwick J., Morton S., Phillips E., Zhang Y., Volkov V. (2008). Parallel Computing Experiences with CUDA. IEEE Micro.

[25] Priimak D. (2014). Finite difference numerical method for the superlattice Boltzmann transport equation and case comparison of CPU(C) and GPU(CUDA) implementations. J. Comput. Phys..

[26] Valsalan P., Sriramakrishnan P., Sridhar S., Latha G.C.P., Priya A., Ramkumar S., Singh A.R., Rajendran T. (2020). Knowledge based fuzzy c-means method for rapid brain tissues segmentation of magnetic resonance imaging scans with CUDA enabled GPU machine. J. Ambient. Intell. Humaniz. Comput..

[27] Gavali P., Banu J.S., Sangaiah A.K. (2019). Chapter 6-Deep Convolutional Neural Network for Image Classification on CUDA Platform. Deep Learning and Parallel Computing Environment for Bioengineering Systems.

[28] Pereira S., Pinto A., Alves V., Silva C.A. (2016). Brain Tumor Segmentation Using Convolutional Neural Networks in MRI Images. IEEE Trans. Med. Imaging.

[29] Ferreira M.R., Torres H.R., Oliveira B., Gomes-Fonseca J., Morais P., Novais P., Vilaca J.L. Comparative Analysis of Current Deep Learning Networks for Breast Lesion Segmentation in Ultrasound Images. Proceedings of the 2022 44th Annual International Conference of the IEEE Engineering in Medicine & Biology Society (EMBC).

[30] Ribeiro R.F., Gomes-Fonseca J., Torres H.R., Oliveira B., Vilhena E., Morais P., Vilaca J.L. Deep learning methods for lesion detection on mammography images: A comparative analysis. Proceedings of the 2022 44th Annual International Conference of the IEEE Engineering in Medicine & Biology Society (EMBC).

[31] Stevens W.R., Wright G.R. (2001). TCP/IP Illustrated (3 Volume Set).

[32] Kurose J.F., Ross K.W. (2010). Computer Networking: A Top-Down Approach.

[33] Bhatt A.A., Whaley D.H., Lee C.U. (2021). Ultrasound-Guided Breast Biopsies. J. Ultrasound Med..

[34] Shaw R. (1987). Vector cross products in n dimensions. Int. J. Math. Educ. Sci. Technol..

[35] Bluvol N., Kornecki A., Shaikh A., Fernandez D.D.R., Taves D.H., Fenster A. (2009). Freehand Versus Guided Breast Biopsy: Comparison of Accuracy, Needle Motion, and Biopsy Time in a Tissue Model. Am. J. Roentgenol..

[36] Rodríguez-Abad C., Fernández-de-la Iglesia J.d.C., Martínez-Santos A.E., Rodríguez-González R. (2021). A Systematic Review of Augmented Reality in Health Sciences: A Guide to Decision-Making in Higher Education. Int. J. Environ. Res. Public Health.

[37] Parekh P., Patel S., Patel N., Shah M. (2020). Systematic review and meta-analysis of augmented reality in medicine, retail, and games. Vis. Comput. Ind. Biomed. Art.

